# A SEPALLATA MADS-Box Transcription Factor, SlMBP21, Functions as a Negative Regulator of Flower Number and Fruit Yields in Tomato

**DOI:** 10.3390/plants13101421

**Published:** 2024-05-20

**Authors:** Jianling Zhang, Tingting Dong, Zongli Hu, Jing Li, Mingku Zhu, Guoping Chen

**Affiliations:** 1 Laboratory of Plant Germplasm Resources Innovation and Utilization, School of Life Sciences, Liaocheng University, Liaocheng 252000, China; zhangjianling0520@126.com; 2 Institute of Integrative Plant Biology, School of Life Science, Jiangsu Normal University, Xuzhou 221008, China; dtt@jsnu.edu.cn (T.D.); mingkuzhu007@126.com (M.Z.); 3 Laboratory of Molecular Biology of Tomato, Bioengineering College, Chongqing University, Chongqing 400030, China; huzongli71@163.com (Z.H.); micy180605@163.com (J.L.)

**Keywords:** MADS-box, *SlMBP21*, meristems, fruit yields, tomato

## Abstract

MADS-box transcription factors act as the crucial regulators in plant organ differentiation. Crop yields are highly influenced by the flower number and fruit growth. However, flower identification is a very complex biological process, which involves many cascade regulations. The molecular mechanisms underlying the genetic regulation of flower identification in cultivated plants, such as tomato, are intricate and require further exploration. In this study, we investigated the vital function of a SEPALLATA (SEP) MADS-box gene, *SlMBP21*, in tomato sympodial inflorescence meristem (SIM) development for the conversion from SIMs to floral meristems (FMs). *SlMBP21* transcripts were primarily accumulated in young inflorescence meristem, flowers, sepals, and abscission zones. The Ailsa Craig (AC^++^) tomato plants with suppressed *SlMBP21* mRNA levels using RNAi exhibited a large increase in flower number and fruit yields in addition to enlarged sepals and inhibited abscission zone development. Scanning electron microscopy (SEM) revealed that the maturation of inflorescence meristems (IMs) was repressed in *SlMBP21*-RNAi lines. RNA-seq and qRT-PCR analyses showed that numerous genes related to the flower development, plant hormone signal transduction, cell cycle, and cell proliferation et al. were dramatically changed in *SlMBP21*-RNAi lines. Yeast two-hybrid assay exhibited that SlMBP21 can respectively interact with SlCMB1, SFT, JOINTLESS, and MC, which play key roles in inflorescence meristems or FM development. In summary, our data demonstrate that *SlMBP21* functions as a key regulator in SIM development and the conversion from SIMs to FMs, through interacting with other regulatory proteins to control the expression of related genes.

## 1. Introduction

The generation of flowers and inflorescences always have great influences on the reproduction of plant progeny and crop yields. Agricultural productivity is strongly influenced by the number of flowering branches, flower number in each inflorescence, and the abscission of flowers or fruits during development, both before and at maturity. The generation of flowers in higher plants is a complex genetic and biochemical process which is divided into at least three steps: the flowering transition, the individual flower initiation, and the floral patterning [1]. During this program, the shoot apical meristems (SAMs), a dome of self-renewing cells located at the shoot apex, first generate leaves before transitioning to inflorescence meristems (IMs), which give rise to lateral (axillary) meristems that either directly differentiate into flowers or transition into flower-bearing shoots [2,3]. In monopodial plants, such as rice and *Arabidopsis*, the SAM is indeterminate and the IM continuously generates floral meristem (FM) laterally, bringing about a finite range of simple inflorescence architectures [4,5]. Whereas, in sympodial plants, such as in tomato, the SAM is determinate and terminated by an inflorescence after 6–12 leaves have been produced and the growth of plants will continue from the bud of axillae at the top leaf [6,7]. Tomato plants continue growth from the generation of a specialized axillary meristem. The axillary meristem grows in the axillary of the last leaf and terminates after generating three leaves. Then, a new axillary meristem is produced in the axil of the last leaf which is generated by the previous axillary meristem. This process is repeated and finally all subsequent axillary meristems generate three leaves, an inflorescence, and the next axillary meristem. These inflorescences are produced by inflorescence meristems (namely, sympodial inflorescence meristems (SIMs) in tomato), which generate a new SIM and the founder cells that generate FM. Each new SIM grows perpendicularly to the previous one, leading to the zigzag pattern of inflorescences in tomato. Finally, each FM forms the floral organs, i.e., sepals, petals, stamens, and pistils [2,8]. 

In wild species of tomato, inflorescences have multiply branched architectures and produce dozens of flowers, while in most cultivated tomatoes, the inflorescences produce several flowers arranged in a zigzag branching pattern [8]. Several mutants with compound numerous or single flowers have been reported in tomato. The *uf* (*uniflora*) mutant, for example, generates fertile, normal, and solitary flowers with a delayed flowering time [9], and plants with *SFT* (*SINGLE FLOWER TRUSS*) loss-of-function alleles only generate a few flowers prior to reverting to vegetative branch architectures and flower later than normal [10]. The mutation of *TMF* (*TERMINATING FLOWER*) in tomato results in an earlier flowering time and the generation of single-flower primary inflorescence with enlarged leaf-like sepals [11]. In contrast, the *s* mutant bears hundreds of flowers due to the loss of function of a homeobox protein encoded by the *S* (*COMPOUND INFLORESCENCE*, the homolog of *WUSCHEL-RELATED HOMEOBOX 9*) gene [8,12], while the shoots of the *an* mutant terminate in cauliflower-like tissues as a result of losing the function of *AN* (an F-box gene, the homolog of *UNUSUAL FLORAL ORGANS*) [13]. Mutation of an APETALA2 (AP2) family member *TARGET OF EAT1* (*SlTOE1*) in tomato results in defective floral organs and plenty of flowers [14]. Although many regulators have been reported to participate in tomato IM and FM development, there are still so many related regulators with unknown functions that need to be investigated. This will not only contribute to enriching the regulatory networks of IM and FM development, but also to further revealing the molecular mechanisms of IM and FM development regulation.

MADS-box proteins are a group of important transcription factors which have been duplicated and diversified extensively during angiosperms evolution, playing vital functions in diverse developmental processes of plants [15], including abiotic stress response, floral organ identity, flowering time, and the regulation of fruit ripening [16,17,18]. For example, *OsMADS26* has multiple functions in regulating various stress responses in rice [19]. Tomato *SlMBP8* acts as a negative regulator in the salt and drought stress [20]. The *Arabidopsis AtAGL21* positively regulates the lateral root development and *AtAGL21* overexpression in plants generates longer and more lateral roots [21]. The MADS-box gene *TAGL1* plays a significant role in the regulation of cuticle development in tomato [22,23]. In *Arabidopsis*, five MADS-box genes, *SVP* (*SHORT VEGETATIVE PHASE*), *AP1* (*APETALA1*), *AGL24* (*AGAMOUS-LIKE GENE 24*), *SOC1* (*SUPPRESSOR OF OVEREXPRESSION OF CONSTANS1*), and *FLC* (*FLOWERING LOCUS C*) participate in regulating flowering time [24,25,26,27,28,29]. Numerous MADS-box genes have been reported to function as the key regulator in the floral organ identity in flowering plants, such as *Arabidopsis AP1/2/3* (*APETALA1/2/3*), *AG* (*AGAMOUS*), *PI* (*PISTILLATA*) [30,31,32,33,34,35] and *TM6*, *TAP3*, *TOMATO AGAMOUS 1* (*TAG1*) in tomato [36,37]. TAP3 and TM6 play vital roles in the regulation of stamen and petal development [36], and TAG1 (TOMATO AGAMOUS 1) functions as an important regulator in the determination of carpels and stamens [37]. In addition, multiple MADS-box genes are also proven to have vital functions in the regulation of fruit development or ripening in tomato, such as *RIN*, *SlMADS1*, *SlMBP3*, *FUL1* (*FRUITFULL1*) and *FUL2* (*SlMBP7*), and so on [38,39,40,41]. 

In addition, some MADS-box genes are reported to regulate the determination of flower number and inflorescence development. For instance, LFY is a key regulator for the establishment of the FM regulatory network [42]. Two *Arabidopsis* genes, *APETALA1* and *CAULIFLOWER*, have redundant roles in the formation of FM [43]. The *AGL6*-like MADS-box gene in wheat is a target of spikelet meristem development regulation and is a key regulator of floral organ identity [44]. Overexpression of two *SHORT VEGETATIVE PHASE* (*SVP*) MADS-box genes *OsMADS22* and *OsMADS55* in rice results in increased flower numbers via the repression of meristem phase transition [45]. In tomato, four MADS-box genes, *TM3*, *STM3*, *FUL2,* and *SlMBP20,* are reported to regulate tomato inflorescence development; the *ful2mbp20* mutants display the increased inflorescence branches with a large number of flowers [46,47]. STM3 is also reported to regulate flower number by regulating the inflorescence meristem via activating *FRUITFULL1*, and overexpression of *STM3* results in highly branched inflorescences with a large flower number [48]. The *j* mutant displays the determinacy loss of meristem which generates a transformation to vegetative growth after two or three flowers were produced [49]. The *MACROCALYX* (*MC*) gene is proven to regulate meristem determinacy and sepal development in tomato; the *vegetative inflorescence* (*mc-vin*) mutant which is generated by a T-DNA insertion into *MC* gene promoter region exhibits a transition to the vegetative growth after generating a few flowers [40,50].

The flower number which is controlled by the determination of cell fate in inflorescence meristems (IM) is a yield-related trait. In angiosperms, the IM produces the founder cells to generate FMs, which form floral organs, including sepals, petals, stamens, and pistils. Therefore, the developmental progression of flowers is vital for crop yield production. Investigations of the molecular mechanisms underlying the development of flowers are essential for the further understanding of reproductive organ formation and improving yield traits. As a sympodial plant, tomato displays the highly structural plasticity of inflorescences. However, the mechanisms underlying the genetic regulation of SIM development and the conversion from SIMs to FMs remain to be further investigated in tomato. Previous studies have reported that *SlMBP21* participates in the regulation of flower abscission zone formation, sepal size, and reproductive development [51,52,53]. In this study, we investigated the vital functions of an SEP MADS-box gene, *SlMBP21*, in SIM development and the conversion from SIMs to FMs. Its transcripts were primarily accumulated in inflorescences, flowers, sepals, and abscission zone. Suppression of *SlMBP21* in the Ailsa Craig (AC^++^) tomato plants using the RNAi method resulted in large increases in flower number and fruit yields, and continuous production of SIM, compared with the wild type. This implies the potential functions of *SlMBP21* in the regulation of flower number and fruit yield through regulating SIM development and the conversion from SIMs to FMs. Then, we carried out further functional investigations of *SlMBP21* via the phenotypic and molecular characterizations of *SlMBP21*-RNAi lines to deeply investigate its functions and molecular mechanisms in the regulation of tomato SIM development and the conversion from SIMs to FMs.

## 2. Methods

### 2.1. Plant Materials and Growth Conditions

In this study, a near-isogenic tomato line, *Solanum lycopersicon* Mill. cv. Ailsa Craig (AC^++^), was used as the wild type plants. The transgenic and WT plants were grown in a standard greenhouse (16 h-day/8 h-night cycle, 25/18 °C day/night temperature, 80% humidity). All the harvested samples were immediately frozen in liquid nitrogen and stored at −80 °C until further use.

### 2.2. SlMBP21 Isolation and Sequence Analysis

Total RNA of tomato inflorescences was obtained using plant RNA kit (OMEGA, Norwalk, CT, USA) following the manufacturer’s instructions. Then, the first strand cDNA was synthesized using 1 µg total RNA with the M-MLV reverse transcriptase (Promega, Madison, WI, USA) and Oligo d(T)_20_ primer. The full length of *SlMBP21* was cloned using primers of *SlMBP21-full*-F/R (Table S1) with 1–2 µg cDNA as the template. After being tailed, the amplified products were inserted into the pMD18-T vector (TaKaRa, Beijing, China). The PCR was performed to pick out the positive clones, and then sequencing was carried out to further confirm the obtained positive clones.

### 2.3. Phylogenetic Analysis

Multiple alignment of SlMBP21 protein with other MADS-box proteins was carried out by DNAMAN (V 6.0). A phylogenetic tree was obtained by MEGA 11 using the neighbor-joining method with the following parameters: Poisson model, pairwise deletion, and bootstrap analysis of 1000 replicates. 

### 2.4. Construction of SlMBP21-RNAi Vector and Plant Transformation

To suppress the expression of the *SlMBP21* gene, RNA interference (RNAi) vectors were constructed. A 381-bp C-terminal specific fragment of *SlMBP21* was obtained using the primers *SlMBP21*-i-F/R (Table S1). Then, the amplified fragments were digested using the restriction enzyme *Kpn* I/*Xho* I and *Hin*d III/*Xba* I (TaKaRa, Beijing, China). After being purified, the digested *SlMBP21* fragments were cloned into the pHANNIBAL vector at *Kpn* I/*Xho* I restriction site and at *Hin*d III/*Xba* I restriction site, respectively. Finally, the needed expression unit including the CaMV (cauliflower mosaic virus) 35S promoter, the sense-orientated *SlMBP21* fragment, *PDK* intron, the antisense-orientated *SlMBP21* and *OCS* terminator, was digested by *Sac* I/*Spe* I and inserted into pBIN 19 at the *Sac* I and *Xba* I site to produce the *SlMBP21*-RNAi vector.

The *SlMBP21*-RNAi binary plasmids were transferred into the *Agrobacterium* LBA4404 strain using the *Agrobacterium*-mediated transformation following the protocols reported by Chen et al. [54]. After the transgenic plants were obtained. The PCR was performed using the primers *NPTII-F*/R (Table S1) to select the positive transgenic plants which will be used for further investigation.

### 2.5. Morphological Observations and Statistic Analysis

The number of flowers and fruits, the average weight of each fruit, and the total weight of all fruits in each inflorescence were statistically analyzed, respectively. At least 10 plants of each line were used to perform each statistical analysis. For scanning electron microscope (SEM), the second inflorescences of wild type and *SlMBP21*-RNAi lines were fixed and prepared. SEM was performed according to Molinero-Rosales et al. [10]. Paraffin sectioning was used to perform the morphological observations of abscission zone of breaker stage fruits by light microscopy following our previous method [41].

### 2.6. RNA-Seq and Data Analysis

The inflorescence meristems (IMs, it is called SIMs in tomato) and floral meristems (FMs) from the second inflorescences of WT and *SlMBP21*-RNAi plants were harvested from at least 10 plants using the dissecting microscope to carry out RNA-seq analysis. Then, the RNA-seq was performed following the same method in our previous study [41]. The sequencing data can be found in the NCBI Sequence Read Archive (SRA, http://www.ncbi.nlm.nih.gov/Traces/sra accessed on 1 April 2024) with accession numbers SAMN40557986–SAMN40557989.

### 2.7. Quantitative Real-Time PCR (qRT-PCR) Analysis

The harvested SIMs and FMs from the second inflorescences of WT and *SlMBP21*-RNAi plants that were used for RNA-seq were also used for the qRT-PCR analysis. Total RNA of each sample of WT and *SlMBP21*-RNAi lines was obtained using plant RNA kit (OMEGA, Norcross, GA, USA). The qRT-PCR analysis was performed using the same method as our previous study [55]. The primers *q*-*SlMBP21-F*/*R* (Table S2) were used to examine the expression levels of *SlMBP21* in WT and *SlMBP21*-RNAi lines. Furthermore, the expression levels of numerous genes were determined simultaneously, including inflorescence meristem fate-related genes *SP* (NM_001247045), *STM3* (XM_010317148), *JOINTLESS* (NM_001319841), and *S* (NM_001247143), cell cycle-related genes *cycA2* (NM_001246839), *cycD3* (NM_001246865), *cdkB2* (NM_001246976), and *E2FE* (XM_004236104), cell wall modification-related genes *cel2* (NM_001247938), *XTH1* (NM_001246929), *EXPA18* (NM_001247832), and *MAN1* (NM_001247646), cytokinin synthesis and degradation-related genes *LOG3* (XM_004246608), *LOG6* (NM_001257982), *CKX2* (NM_001257980), and *CKX7* (NM_001257979), auxin synthesis and response-related genes *IAA2* (XM_010324706), *IAA5* (NM_001279133), *ARF5* (NM_001247616), and *ARF9* (NM_001247605), and ethylene synthesis and response-related genes *ACS1A* (NM_001246993.1), *ACS6* (NM_001247235.1), *ERF*-*H1* (NM_001247919), and *ERF5* (NM_001330445). Primers are shown in Supplementary Table S2. Three independent biological repeats were performed.

### 2.8. Yeast Two-Hybrid Assay

The ORFs of *SlMBP21*, *SFT*, *SlCMB1*, *JOINTLESS*, and *MC* were amplified by PCR using the primers *SlMBP21* (Y2H)*-F*/R, *SFT* (Y2H)*-F*/*R*, *SlCMB1* (Y2H)*-F*/*R*, *JOINTLESS* (Y2H)*-F*/*R*, and *MC* (Y2H)*-F*/*R* (Table S1), respectively. The PCR products of each gene were respectively digested with restriction enzymes *Eco*R I and *Bam*H I (TaKaRa, China). After that, *SlMBP21* was linked into the *Eco*R I/*Bam*H I site of the pGBKT7 to obtain the pGBKT7-*SlMBP21* vector. Simultaneously, the ORFs of *SFT*, *SlCMB1*, *JOINTLESS,* and *MC* were digested with restriction enzymes *Eco*R I and *Bam*H I and inserted into the *Eco*R I/*Bam*H I site of the pGADT7 to obtain the pGADT7-*SFT*, pGADT7-*SlCMB1*, pGADT7-*JOINTLESS,* and pGADT7-*MC* vector, respectively. Then, pGBKT7-*SlMBP21* vector was transferred into Y2HGold. The pGADT7-*SFT*, pGADT7-*SlCMB1*, pGADT7-*JOINTLESS,* and pGADT7-*MC* vectors were transferred into Y187, respectively. The yeast two-hybrid assays were performed using the same method as described in our previous study [55].

## 3. Results

### 3.1. Molecular Characterization of SlMBP21

**Figure 1:**
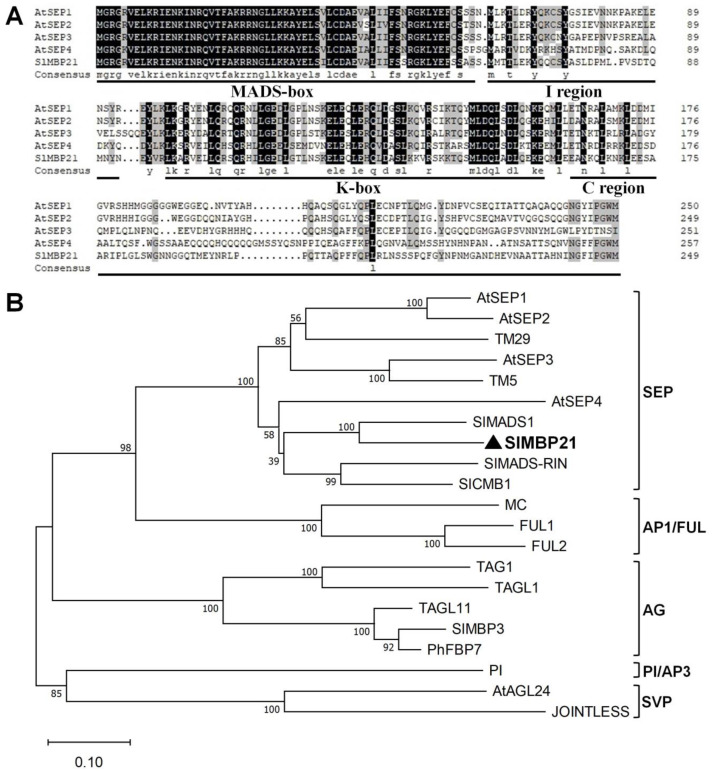
**Multiple sequence alignment and phylogenetic analysis of SlMBP21 and other known MADS-box proteins.** (**A**) Multiple sequence alignment of SlMBP21 and other MADS-box proteins. Identical amino acids are shaded in black, and similar amino acids are shaded in gray. The MADS-box, K-box, I region, and C region are identified. (**B**) Phylogenetic analysis of the SlMBP21 and other known MADS-box proteins. SlMBP21 is indicated by bold and black triangle. Accession numbers and corresponding references for the proteins listed are as follows: AtSEP1 (AED92207.1), AtSEP2 (AEE73791.1), TM29 (NP_001233911), AtSEP3 (AEE30503.1), TM5 (NM_001247455), AtSEP4 (NM_126418), SlMADS1 (NM_001247451), SlMBP21 (NM_001288650), SlMADS-RIN (NM_001247741), SlCMB1 (XM_004237013), MC (NM_001247736.1), FUL1 (XM_019214141), FUL2 (NM_001307938), TAG1 (NM_001279252), TAGL1 (AY098735), TAGL11 (NM_001247265), SlMBP3 (XM_004241858), PhFBP7 (X81651), PI (LOC101265756), AtAGL24 (NM_118587), JOINTLESS (NM_001319841). The numbers at the nodes indicate the bootstrap values. The bar at the bottom indicates the relative divergence of the sequences examined.

To study the genetic regulation of inflorescence architecture in tomato (*Solanum lycopersicum*), we concentrated our functional investigations on the SEP MADS-box member *SlMBP21*. Alignment analysis of MADS-box protein sequences showed that *SlMBP21* had the conserved domains (namely MADS domain, I domain, and K domain) and its C-terminal region was highly different than other sequences of MADS-box proteins (Figure 1A). Moreover, phylogenetic analysis exhibited that *SlMBP21* belonged to the *SEPALLATA* (*SEP*) clade (Figure 1B).

### 3.2. Expression Patterns of SlMBP21 in Different Tomato Tissues 

**Figure 2:**
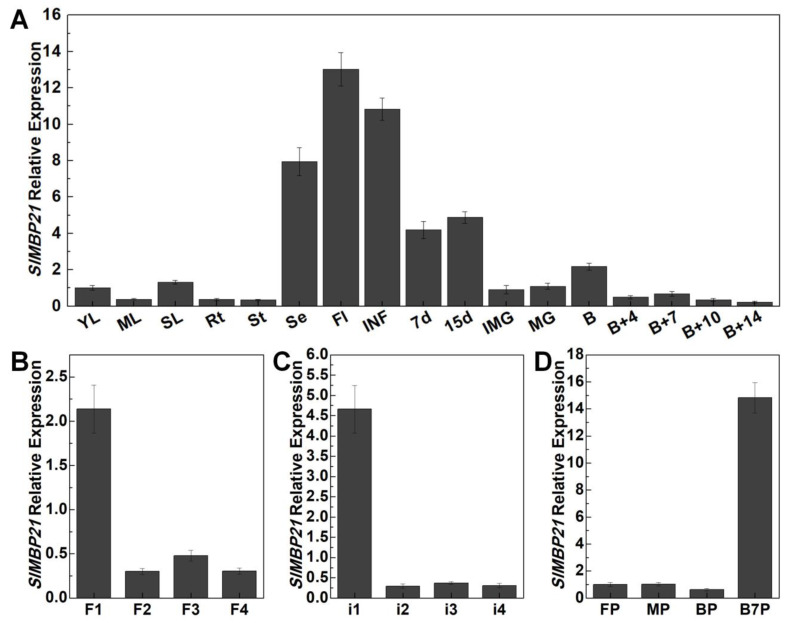
**Expression profile of *SlMBP21* in wild-type tissues.** (**A**) The expression of *SlMBP21* in Rt, roots (a mixture of various roots of various ages); St, stems (a mixture of various periods stems); YL, young leaves; ML, mature leaves; SL, senescent leaves; Se, sepals; Fl, flowers at anthesis; INF, the sympodial inflorescence meristems (SIMs) and the floral meristems (FMs) in inflorescences; IMG, immature green fruits; MG, mature green fruits; B, breaker fruits; B + 4, 4 days after breaker fruits; B + 7, 7 days after breaker fruits, B + 10, 10 days after breaker fruits; B + 14, 14 days after breaker fruits in wild type. (**B**) The expression of *SlMBP21* in flowers of wild type. F1, 1–3 mm flowers; F2, 4–6 mm flowers; F3, 7–9 mm flowers; F4, flowers which are fully opened and greater than 10 mm in length. (**C**) The expression of *SlMBP21* in inflorescence and floral meristems of wild type. i1, inflorescence and floral meristems with F1 stage flowers; i2, inflorescence and floral meristems with F2 stage flowers; i3, inflorescence and floral meristems with F3 stage flowers; i4, inflorescence and floral meristems with F4 stage flowers; (**D**) The expression of *SlMBP21* in pedicel abscission zones. FP, abscission zone of flower pedicel; MP, abscission zone of MG stage fruit pedicel; BP, abscission zone of breaker stage fruit pedicel; B7P, abscission zone of B + 7 stage fruit pedicel. Vertical bars represent SE.

To understand the potential functions of *SlMBP21* in tomato development, the expression levels of *SlMBP21* in roots, stems, leaves, sepals, and a series of developmental stages of flowers inflorescences and fruits were analyzed. Low-level expression of *SlMBP21* was observed in vegetative organs such as roots, stems, leaves, and B + 4 (4 days after breaker fruits) to B + 14 fruits (14 days after breaker fruits) (Figure 2A). In all detected tissues, *SlMBP21* was transcribed more abundantly in sepals, flowers, inflorescences, and 7d to B (breaker) fruits (Figure 2A). In the different developmental stages of flowers and inflorescences, *SlMBP21* transcripts were preferentially accumulated at the onset of inflorescence production and young flowers, and decreased with the development of flowers and inflorescences (Figure 2B,C). Furthermore, *SlMBP21* transcript accumulation was detected at low levels in the early stage of pedicel abscission zone formation, and increased dramatically in the pedicel abscission zones of B + 7 (7 days after breaker fruits) stage fruits (Figure 2D). These results indicate that *SlMBP21* may have vital functions in the development of inflorescences, flowers, sepals, and pedicel abscission zone.

### 3.3. SlMBP21-RNAi Lines Exhibited Abundant Flower Production and Increased Fruit Yield

**Figure 3:**
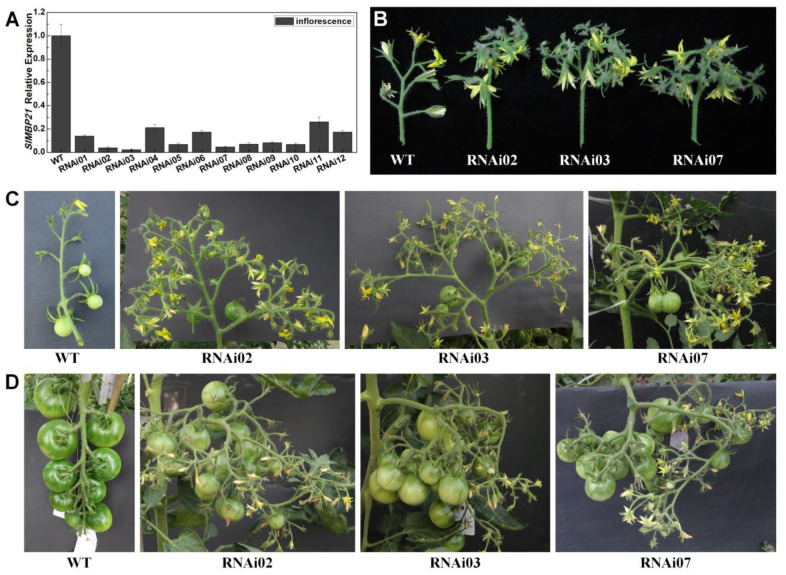
**Gene expression analyses of *SlMBP21* and phenotype of flower number and fruit yield in WT and *SlMBP21*-RNAi lines.** (**A**) The relative expression of *SlMBP21* in WT and 12 *SlMBP21*-RNAi lines. The tissue examined was inflorescences with meristem and no flowers. Expression values are relative to the *SlCAC* gene. (**B**) Phenotypes of flower number (unopened and opened flowers) and floral meristem in WT and *SlMBP21*-RNAi lines. (**C**) Phenotypes of IMG fruits and flowers (unopened and opened flowers) in WT and *SlMBP21*-RNAi lines. (**D**) Phenotypes of MG fruits in WT and *SlMBP21*-RNAi lines. In this stage, the WT inflorescence has had no flowers but the *SlMBP21*-RNAi lines can unceasingly produce flowers (unopened and opened flowers). The WT plants always produce about 7–10 flowers and 6–9 fruits, while the *SlMBP21*-RNAi lines can produce hundreds of flowers but only part of them can successfully produce fruits due to the insufficient nutrition. However, the number of fruits of transgenic plants is still significantly more than that of wild type.

To further study *SlMBP21* functions in tomato development, we constructed the *SlMBP21*-RNAi vector (Figure S1) and transformed it into wild-type tomato plants by *Agrobacterium tumefaciens*-mediated T-DNA transfer. Finally, twelve independent RNAi lines in which the transcripts of *SlMBP21* were dramatically decreased were obtained, and three lines (RNAi02, RNAi03, RNAi07) which displayed the *SlMBP21* lowest expression levels were selected for the further investigations (Figure 3A). Then, the expression of *SlMBP21* was also detected in sepals and the abscission zone of the RNAi02, RNAi03, and RNAi07 lines, respectively. The results exhibited that *SlMBP21* transcripts were markedly reduced in sepals and the abscission zone of these three lines, respectively (Figure S2A,B).

**Figure 4:**
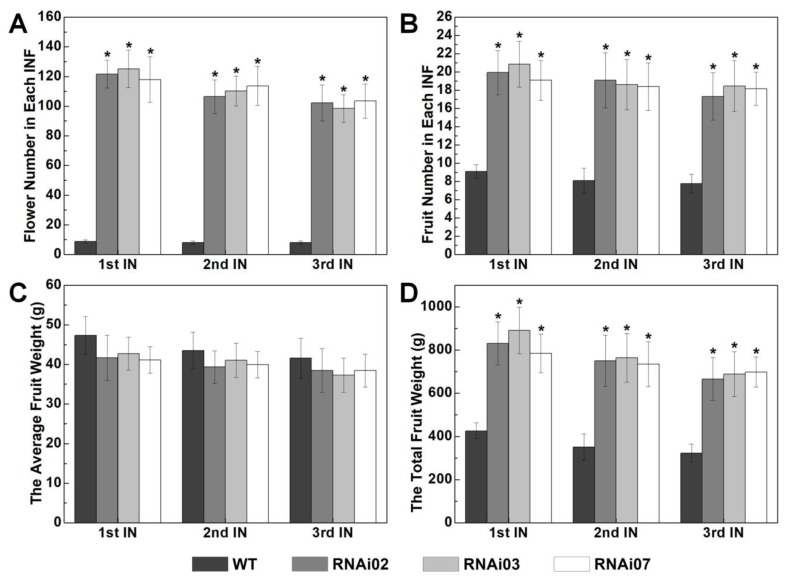
**Statistic analysis of flower and fruit number, average fruit weight, and total fruit weight in each inflorescence.** (**A**) The flower number in each inflorescence of WT and *SlMBP21*-RNAi lines. (**B**) The fruit number in each inflorescence of WT and *SlMBP21*-RNAi lines. (**C**) The average fruit weight of each fruit in each inflorescence of WT and *SlMBP21*-RNAi lines. (**D**) The total fruit weight in each inflorescence of WT and *SlMBP21*-RNAi lines. First IN, the first inflorescence; second IN, the second inflorescence; third IN, the third inflorescence. The inflorescence at the bottom is recorded as the first inflorescence from the bottom to the top. The significant differences between the WT and RNAi lines were marked with the asterisks (*p* < 0.05). All data are means (±SE) of three independent biological replicates and at least 12 plants of each line were used to perform statistical analysis of each biological replicate.

After being suppressed, we found that the RNAi lines generated abundant flowers and more fruits. It seems that the SIMs in inflorescences of *SlMBP21*-RNAi lines were always in the state of continuous and vigorous differentiation, while the SIMs of wild-type plants stopped differentiation after producing several flowers. As a result, the RNAi lines generated abundant flowers and more fruits apart from enlarged sepals and inhibited abscission zone formation, while the WT plants generated a normal number of flowers, sepal size, and abscission zone (Figure 3B,C and Figure S2C–H). Then, the flower numbers of the first, second, and third inflorescences of *SlMBP21*-RNAi plants and wild-type plants were counted. The results showed that there were about 7–10 flowers in one inflorescence of WT plants, while the *SlMBP21*-RNAi plants can possess over 100 flowers in each inflorescence (Figure 4A). Finally, we found that although the *SlMBP21*-RNAi lines can produce hundreds of flowers, not every flower can successfully complete fruit setting and produce fruits. This may be due to the fact that too many flowers needed more nutrition, which eventually resulted in insufficient nutrition to supply so many flowers to complete the fruit setting. However, the average fruit number of each inflorescence in *SlMBP21*-RNAi lines was still significantly higher than that in WT plants (Figure 4B). Furthermore, another statistic analysis exhibited that although the average weight per fruit in each inflorescence (Figure 4C) was reduced, the total weight of all fruits in each inflorescence was dramatically higher than that in WT plants (Figure 4D). These data together demonstrate that *SlMBP21* has vital functions in controlling floral meristem development, flower production and fruit yield in tomato.

### 3.4. Maturation of SIMs Was Repressed in SlMBP21-RNAi Lines

**Figure 5:**
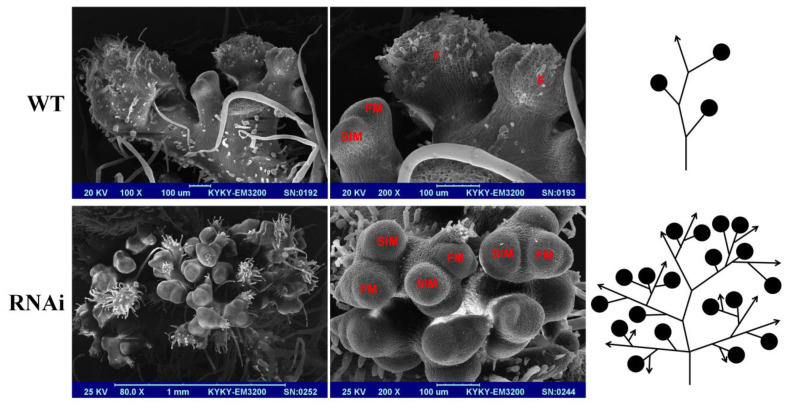
**Scanning electron micrographs (SEMs) and schematic diagrams of meristem development in WT and *SlMBP21*-RNAi lines.** Scanning electron micrographs and schematic diagram of meristem development in WT and *SlMBP21*-RNAi lines. The SEM figures indicate the number and details of flowers, SIM, and FM under different magnifications. The right schematics reflect inflorescence with SIM, FM, and flowers in the branches of WT and *SlMBP21*-RNAi lines. A solid black circle indicates flower or FM. Line arrow indicates SIM. F, flower; FM, floral meristem; SIM; sympodial inflorescence meristem.

Tomato inflorescences are compound shoots, resulting from sympodial inflorescence meristems (SIMs, i.e., IMs), each of which gives rise to another SIM before failure of the meristems emerging from the SIM to specify floral identity [8]. The rate of SIM maturation drives inflorescence complexity and flower production, and a delay in SIM maturation can increase the complexity of inflorescence [56,57]. Finally, the SIMs terminate in the last FMs and no new SIM is produced after 6–10 flowers are produced. However, in our study, the scanning electron microscopy (SEM) analysis showed that the inflorescences in *SlMBP21*-RNAi lines can simultaneously give rise to multiple SIMs before terminating in one FM (Figure 5). These SIMs can further generate more FMs and finally produce a very large number of flowers (Figure 5). These results reveal that the conversion from SIMs to FMs is delayed in *SlMBP21*-RNAi lines, which is regarded as the immediate reason of the extreme branched inflorescences. These data suggest that *SlMBP21* is a crucial regulator in the differentiation and maturation of SIMs.

### 3.5. Transcriptome Analysis in Meristems of the WT and SlMBP21-RNAi Lines

**Figure 6:**
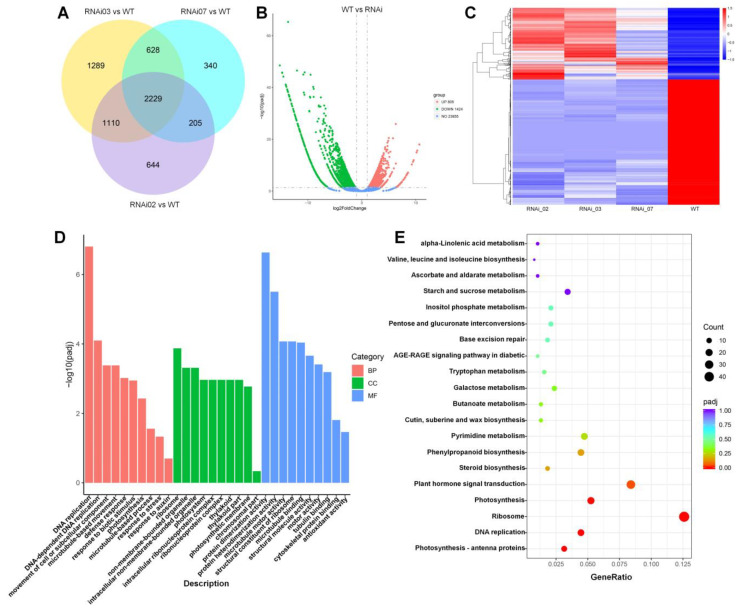
**RNA-seq analysis of genes regulated by SlMBP21 during tomato meristem development.** (**A**) Venn diagram represents the co-expressed DEGs between WT and *SlMBP21*-RNAi lines. Overlapping regions of three circles indicate DEGs that are expressed simultaneously in WT and *SlMBP21*-RNAi lines. The number only in one circle represents DEGs that are only expressed in WT or *SlMBP21*-RNAi lines. (**B**) Volcano plot visualizing the differentially expressed genes (DEGs). The DEGs are shown in red and green. The x-axis represents the fold change in WT vs. RNAi (on a log2 scale), and the y-axis represents the negative -log10-transformed *p*-values (*p* < 0.05) of the *t*-test for finding differences between the samples. (**C**) Hierarchical clustering analysis of DEGs in meristem of RNAi and WT lines. Red and blue colors in the heat maps represent induced and repressed genes, respectively. Scale bar denotes the value of log10(FPKM + 1). FPKM, Fragments Per Kilobase of transcript sequence per Millions base pairs sequenced. (**D**) Gene Ontology (GO) classification. (**E**) The primarily enriched KEGG pathway enrichment of up- and down-regulated DEGs. ‘GeneRatio’ indicates the ratio of the number of differential genes associated with one KEGG pathway to the total number of all DEGs.

Numerous genes of different families have been proven to function as the vital regulators in regulating plant development. To explore the expression changes of genes during meristem (SIM and FM) development in WT and *SlMBP21*-RNAi plants, we performed the RNA-seq analyses. In this RNA-seq, four cDNA libraries including the WT and three RNAi lines were produced to be sequenced. More than 23 million sequence reads were obtained from each cDNA library, representing the sequence data of >7 Gb for each sample. Finally, a total number of 23,855 genes were identified from meristems (SIMs and FMs) of WT and *SlMBP21*-RNAi lines using FPKM > 1 as the standard to determine whether genes were expressed. RNA-seq data of these samples showed good correlations and were appropriate for further study (Figure S3). A summary of RNA-seq, mapping, annotation, and assembly was shown in Table S3. As a consequence, a total number of 2229 genes with 805 genes up-regulated and 1424 genes down-regulated were identified as the differentially expressed genes (DEGs) between WT and *SlMBP21*-RNAi lines using padj < 0.05 and |log2 (fold-change)| ≥ 1 as the significant cut-off value in DESeq2 (Figure 6A–C, Table S4). Among these DEGs, numerous genes belonging to different significant gene families and phytohormone synthesis were identified, such as AUX/IAA, GRAS, ARF, WRKY, Dof, MADS-box, AP2/ERF, bZIP, EXP, bHLH, synthesis and response of gibberellin, auxin and ethylene, and so on.

Gene ontology (GO) functional enrichment analysis of these DEGs was implemented to classify them according to their functions in molecular function (MF), cellular component (CC), and biological process (BP). The results showed that these DEGs were enriched in 638 GO terms (82 CC, 235 MF, 321 BP; padj < 0.05). For CC, DEGs were mainly enriched in ribosome, organelle, photosystem, ribonucleoprotein complex, and so on. For MF, most of the DEGs had something to do with protein activity, protein binding, and cell structural constituent. For BP, DEGs were primarily enriched in DNA replication, cell cycle, cell wall modification, response to stress and hormone, and so on (Figure 6D, Table S5). KEGG analysis displayed that 79 pathways were identified and a majority of these DEGs were primarily enriched in some pathways in connection with plant hormone signal transduction, metabolism, biosynthesis, DNA replication, and MAPK signaling pathway, and so on (Figure 6E, Table S6 KEGG). These data demonstrate that these DEGs participate in regulating tomato SIM and FM development via controlling the transcripts of genes related to cell cycle, enzyme activity, plant hormone signal transduction, and cell proliferation.

### 3.6. Expression Profile Analysis of Genes Related to SIM and IM Development

**Figure 7:**
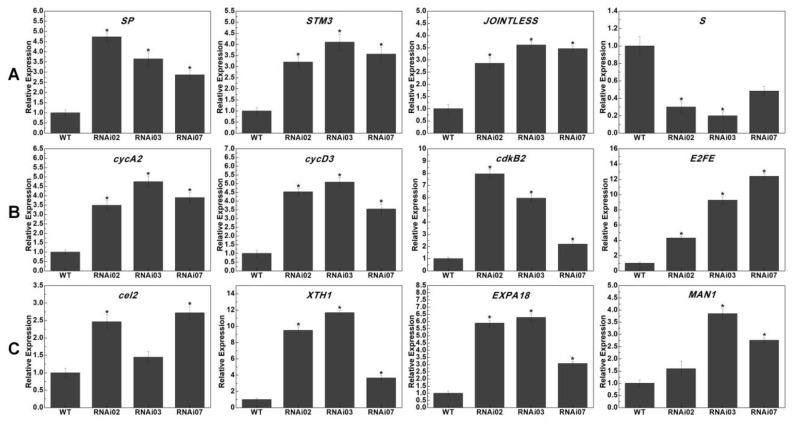
Expression analysis of genes related to inflorescence meristem development, cell cycle, and cell wall modification in meristems of WT and *SlMBP21*-RNAi lines. (**A**) Expression of inflorescence meristem fate-related genes (*SP*, *STM3*, *JOINTLESS*, and *S*) in meristems of WT and *SlMBP21*-RNAi lines. (**B**) Expression of cell cycle-related genes (*cycA2*, *cycD3*, *cdkB2*, and *E2FE*) in meristems of WT and *SlMBP21*-RNAi lines. (**C**) Expression of cell wall modification-related genes (*Cel2*, *XTH1*, *EXPA18*, and *MAN1*) in meristems of WT and *SlMBP21*-RNAi lines. The significant differences between the WT and RNAi lines were marked with the asterisks (*p* < 0.05). Three replications for each sample were performed. All data are means (±SE) of three independent biological replicates.

**Figure 8:**
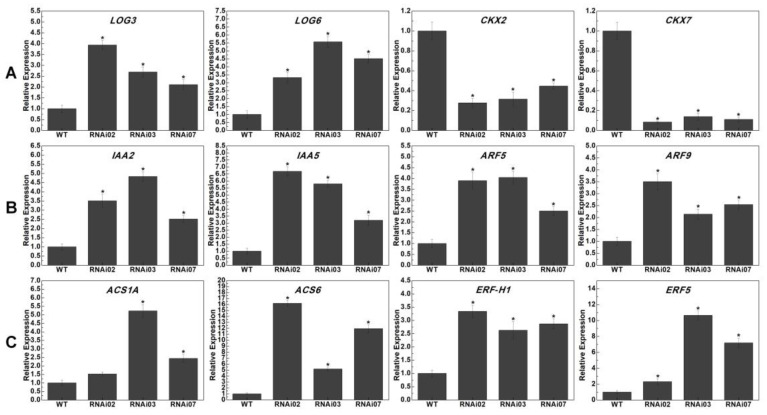
Expression analysis of genes related to plant hormone synthesis and response in meristems of WT and *SlMBP21*-RNAi lines. (**A**) Expression of cytokinin synthesis and degradation-related genes (*LOG3*, *LOG6*, *CKX2*, and *CKX7*) in meristems of WT and *SlMBP21*-RNAi lines. (**B**) Expression of auxin synthesis and response-related genes (*IAA2*, *IAA5*, *ARF5*, and *ARF9*) in meristems of WT and *SlMBP21*-RNAi lines. (**C**) Expression of c ethylene synthesis and response-related genes (*ACS1A*, *ACS6*, *ERF-H1*, and *ERF5*) in meristems of WT and *SlMBP21*-RNAi lines. The significant differences between the WT and RNAi lines were marked with the asterisks (*p* < 0.05). Three replications for each sample were performed. All data are means (±SE) of three independent biological replicates.

To further verify our transcriptome data and explore the vital functions of *SlMBP21* in meristem (SIM and FM) development, some genes which participate in floral meristem development, cell wall metabolism, cell cycle, signal transduction, cell wall modification, and plant hormone metabolism and response were selected from our RNA-seq data to perform the qRT-PCR analysis in the meristems of WT and *SlMBP21*-RNAi plants. The results exhibited that the transcripts of these genes showed varying degrees in meristems (Figure 7 and Figure 8). The transcriptional accumulations of *SP*, *STM3*, and *JOINTLESS* were dramatically increased, whereas *S* was markedly reduced in *SlMBP21*-RNAi lines (Figure 7A). The transcripts of four cell cycle-related genes (*cycA2*, *cycD3*, *cdkB2*, and *E2FE*) were observably increased in *SlMBP21*-RNAi lines (Figure 7B). The expression levels of four genes, *cel2*, *XTH1*, *EXPA18*, and *MAN1*, which were involved in cell wall modification were significantly increased in *SlMBP21*-RNAi lines (Figure 7C). 

Phytohormones, such as cytokinin, auxin, gibberellin, ethylene, and so on play crucial regulatory functions in plant development [58,59,60]. Here, we also detected several genes that are involved in regulating the synthesis, degradation, or responses of three kinds of phytohormones (cytokinin, auxin, ethylene). These phytohormones promote cell development at the early stage of organ development. Our results displayed that the transcripts of two genes (*LOG3* and *LOG6*) related to cytokinin synthesis were significantly up-regulated, while two degradation-related genes (*CKX2* and *CKX7*) were decreased (Figure 8A). The transcripts of auxin synthesis-related genes (*IAA2* and *IAA5*) and auxin response-related genes (*ARF5* and *ARF9*) were obviously increased in *SlMBP21*-RNAi lines (Figure 8B). Moreover, the transcripts of two known genes (*ACS1A* and *ACS6*), which participated in ethylene synthesis, and two ethylene response genes (*ERF-H1* and *ERF5*) were prominently up-regulated in *SlMBP21*-RNAi lines (Figure 8C). These data indicate that *SlMBP21* may regulate SIM development and the switch from SIMs to FMs by either directly or indirectly regulating the transcripts of genes related to meristem development, cell cycle, plant hormone signal transduction, and cell wall modification, and so on.

### 3.7. SlMBP21 Could Interact with SFT, SlCMB1, JOINTLESS, and MC

**Figure 9:**
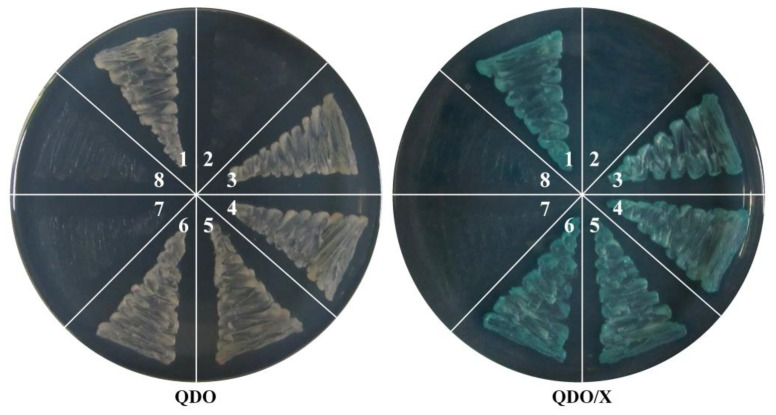
**Yeast two-hybrid assay of SlMBP21 with SFT, SlCMB1, JOINTLESS, and MC Proteins.** QDO, SD medium without Trp, Leu, His, and Ade; QDO/X, SD medium without Trp, Leu, His, Ade and with X-α-Gal. 1. pGBKT7-53 and pGADT7-T (positive control); 2. pGBKT7-Lam and pGADT7-T (negative control); 3. pGBKT7-SlMBP21 and pGADT7-SFT (interaction between SlMBP21 and SFT); 4. pGBKT7-SlMBP21 and pGADT7-SlCMB1 (interaction between SlMBP21 and SlCMB1); 5. pGBKT7-SlMBP21 and pGADT7-JOINTLESS (interaction between SlMBP21 and JOINTLESS); 6. pGBKT7-SlMBP21 and pGADT7-MC (interaction between SlMBP21 and MC); 7. pGADT7 (empty bait vector control); 8. pGBKT7 (empty prey vector control).

Transcription factors usually form homologous or heterologous dimers or polymers with other regulatory proteins to control plant development. To explore the interactions between SlMBP21 and other regulators, SFT, SlCMB1, JOINTLESS, and MC were selected for the yeast two hybrid assay. In tomato, these four proteins have been proven to be the vital regulators during tomato inflorescence development [10,40,61,62]. The open reading frame (ORF) of *SlMBP21* was amplified and cloned into pGBKT7 as the bait and the ORFs of *SFT*, *SlCMB1*, *JOINTLESS*, and *MC* were each inserted into pGADT7 as prey, respectively (Figure S4A,B). Self-activation of pGBKT7-SlMBP21 was tested and a negative result was observed (Figure S5). Moreover, the empty pGBKT7 and pGADT7 vectors were also used as the negative controls, respectively. Figure 9 shows that the yeast can grow on selective media (QDO) and turn blue on X-α-gal indicator plates (QDO/X), demonstrating that SlMBP21 can interact with SFT, SlCMB1, JOINTLESS, and MC, respectively.

## 4. Discussion

The transition from vegetative to reproductive growth is a significant event for flowering plants. In this process, the noticeable changes are the production of flowers. Previous investigations have proven that MADS-box proteins play crucial functions in reproductive development [63,64,65,66]. *SlMBP21* is an SEP MADS-box gene according to the phylogenetic analysis. Its expression patterns in tomato tissues suggest that it may take part in regulating tomato inflorescence development. Then, *SlMBP21*-RNAi lines produced abundant flowers with the enlarged sepals and lacked pedicel abscission zone, which are consistent with previous studies [52,67,68]. In addition, the repressed maturation of SIM and a delay in the conversion from SIMs to FMs were also observed in *SlMBP21*-RNAi lines. These results demonstrate that *SlMBP21* plays indispensable roles in regulating tomato SIM development and the conversion from SIMs to FMs in addition to its functions in the abscission zone and sepal development.

In tomato, multiple genes which play crucial functions in plant inflorescence development have been identified. This will be conducive to the regulatory network investigations of flower formation and meristem (IM and FM) development. For instance, *STM3* regulates inflorescence meristem development by activating *FRUITFULL1*, overexpression of *STM3* results in increased flowers [48]. Overexpression of the *SP* (*SELF-PRUNING*) gene in tomato results in replaced flowers by leaves and suppressed transformation from vegetative to reproductive growth [69]. In *jointless* mutants, after one or two flowers were produced, the inflorescences produce a shoot rather than new flowers [62]. Mutation of the *S* gene in tomato results in highly branched inflorescences with abundant flowers [13]. In this study, the transcripts of *STM3*, *SP*, *JOINTLESS* were increased, whereas the expression of *S* was observably suppressed in *SlMBP21*-RNAi lines based on our RNA-seq and qRT-PCR data. Therefore, increased transcripts of *STM3*, *SP*, *JOINTLESS*, and reduced expression of *S* may lead to the branched inflorescences with dramatically increased flowers in *SlMBP21*-RNAi lines. Based on these results, we speculate that decreased *SlMBP21* transcripts results in the expression changes of some genes relating to inflorescence or meristem development, and finally the *SlMBP21*-RNAi lines produce highly branched inflorescences with significantly increased flowers and fruits.

In the differentiation of flowers from SAM to IM, from the IM (it is named SIM in tomato) to FM, and from the FM to flowers, numerous genes play crucial regulatory roles in these processes, such as genes related to biosynthesis and metabolism of plant hormones, cell wall modification, cell cycle, and other biological processes. In eukaryotes, the cell cycle is principally controlled by heterodimeric complexes of cyclins (CYCs) and cyclin-dependent kinases (CDKs) [70,71]. Pectin lyases (PLs), β-xylosidases (XYLs), endo-1,4-β-glucanases (CELs), β-galactosidases (TBGs), pectin methylesterases (PMEs), polygalacturonases (PGs), expansins (EXPs), and xyloglucan endotransglucosylases/endohydrolases (XTHs) play important regulatory roles in the modification of cell walls [72]. The plant hormones (gibberellins (GAs), IAA, ethylene, cytokinin, et al.) can regulate plant development via tissue elongation or expansion, such as roots, stems, leaves, flowers, and fruits [73,74,75]. During gibberellin (GAs) synthesis, GA20oxs and GA3oxs control the bioactive GAs production, whereas GA2oxs catalyze GAs deactivation to produce inactive GAs [76]. GAs can regulate numerous aspects of plant development, such as the stem elongation, flowers development, pollen maturation, and so on [75]. Auxin response factor ARF5 controls fruit set and development through mediating auxin and gibberellin signaling, and ARF9 regulates cell division during early fruit development in tomato [77,78]. Overexpression of *IAA1* with domain II mutation results in impaired cell division and elongation in *Arabidopsis* [79]. Cytokinin and auxin regulate almost every aspect of plant development, such as the cell division and differentiation, meristem function, senescence and stress responses, and so on. Cytokinin can maintain the stem cell population in the SAM, and auxin can specify the meristematic fate in the SAM [80]. In specific developmental conditions, ethylene can positively influence cell division and expansion. Ethylene and its downstream regulators AtERF018 and AtERF109 promote cell division in the process of stem vasculature development in *Arabidopsis* [81]. In grape berry and *Sagittaria pygmaea*, ethylene increases the transcripts of *XTHs* and promotes cell expansion and cell wall loosening [82,83]. 

In our RNA-seq analysis, the transcripts of DEGs related to cell cycle, enzyme activity, plant hormone signal transduction, cell proliferation, and cell wall modification were significantly increased or decreased. Furthermore, the expression levels of genes related to inflorescence development (*SP*, *STM3*, *JOINTLESS*, and *S*), cell cycle (*cycA2*, *cycD3*, *cdkB2*, and *E2FE*), cell wall modification (*cel2*, *XTH1*, *EXPA2*, and *MAN1*), the metabolism or responses of cytokinin (*LOG3*, *LOG6*, *CKX2*, and *CKX7*), auxin (*IAA2*, *IAA5*, *ARF5*, and *ARF9*), and ethylene (*ACS1A*, *ACS6*, *ERF-H1*, and *ERF5*) were dramatically up- or down-regulated. In addition, our previous study had shown that three ethylene-responsive elements (ERE motif), one auxin-responsive element (TGA-box), and one gibberellin-responsive element (P-box) were found in promoter sequence of *SlMBP21* [68], further suggesting that *SlMBP21* may regulate tomato organ development by affecting plant hormone levels. These results demonstrate that *SlMBP21* may regulate tomato SIM differentiation and the conversion from SIMs to FMs through controlling the transcripts of genes relating to cell development, plant hormone signal transduction and meristem development, and biological processes.

Up to now, MADS-box proteins have been widely proven to play vital functions in regulating plant reproductive development. Previous studies have reported that MADS-box proteins can interact with other regulators to form the dimers or higher-order protein complexes to control plant growth and development [84,85]. *MC* encodes an *AP1*/SQUAMOSA-like MADS-box transcription factor and is an important regulator of inflorescence and sepal development, and also controls tomato fruit abscission zone development by interacting with J [40,86]. The MADS-box protein JOINTLESS functions as an important regulator in inflorescence development and the abscission zone of tomato [49,62]. *JOINTLESS* has also been proposed to be involved in the timing of floral meristem maturation and to reduce the time window for floral meristem maturation, and the *jointless* mutants generate two or three flowers followed by leaves in inflorescences [87]. The *sft* mutant displays the conversion from reproductive to vegetative growth after generating one or several flowers [10,12]. An additional study reports that a target of SFT and JOINTLESS participates in a protein complex to suppress the floral meristem fate [87]. Our previous study showed that the suppression of MADS-box protein *SlCMB1* results in enlarged sepals and longer and loss of determinacy of SIM [61]. It has been reported that MC can interact with JOINTLESS to form a dimer [86], and JOINTLESS can interact with itself to form a homodimer, but MC cannot [52]. Furthermore, SlMBP21, MC, and J can form trimer in vivo [52]. In our study, the yeast two-hybrid assay showed that SlMBP21 can interact with SFT, MC, JOINTLESS, and SlCMB1, respectively. Hence, we further speculate that SlMBP21 may form dimer or trimer with SFT, MC, JOINTLESS, and SlCMB1 to control SIM and FM fate via regulating the expression of related genes. 

## 5. Conclusions

The fate of inflorescence meristem and floral meristem is a very complex biological process, which involves many cascade regulations, and still needs to be further explored. In this study, we evaluated the physiological, molecular, and morphological features of *SlMBP21*-RNAi tomato plants. Although the accurate regulatory mechanisms of *SlMBP21* in tomato SIM development and the conversion from SIMs to FMs is still to be investigated, *SlMBP21* should be a significant target to explore the regulatory mechanisms exploring tomato SIM development. This will provide a new method to improve the fruit yield of tomato plants and other crops. In summary, our data demonstrate that *SlMBP21* functions as the crucial regulator in tomato SIM development and the conversion from SIMs to FMs through interactions with other regulatory proteins to regulate the activities of related regulators or the expression levels of genes relating to numerous biological processes. Identifying the functions of *SlMBP21* in tomato SIM development and the conversion from SIMs to FMs not only extends our understanding of the biological roles of MADS-box proteins in plants but also provides new insights into IM development, an important agronomic trait.

## Data Availability

The data and materials supporting the conclusions of this study are included within the article.

## Supplementary Materials

The following supporting information can be downloaded at: https://www.mdpi.com/article/10.3390/plants13101421/s1, Figure S1: The construction of *SlMBP21*-RNAi vectors; Figure S2: The Phenotype of sepals and abscission zone in *SlMBP21* Repressed Lines; Figure S3: Correlation analysis (through Pearson’s correlation coefficient) of RNA-seq data among WT, RNAi02, RNAi03, RNAi07; Figure S4: Construct of *SlMBP21*, *JOINTLESS*, *MC*, *SlCMB1* and *SFT* gene for the yeast two-hybrid vector; Figure S5: Self-activation assay of pGBKT7-*SlMBP21* in the yeast two-hybrid assay; Table S1: Specific primer sequences used for *SlMBP21* gene amplification and cloning procedures; Table S2: Specific primer sequences used for qRT-PCR analysis; Table S3: The data set summary of transcriptome sequence; Table S4: Total DEGs indetified in this RNA-seq; Table S5: GO enrich of DEGs; Table S6: KEGG enrich of DEGs.
