# Supplementary material for: A SEPALLATA MADS-Box Transcription Factor, SlMBP21, Functions as a Negative Regulator of Flower Number and Fruit Yields in Tomato
Source: Plants (Basel). 2024 May 20;13(10):1421. doi: 10.3390/plants13101421 (PMC11125064; doi:10.3390/plants13101421)
Supplement: Supplementary file 1 [file plants-13-01421-s001.zip › Supplementary files.pdf]

Supplementary information

Table S1. Specific primer sequences used for *SlMBP21* gene amplification and cloning procedures.

Primer Name	Primer Sequence (5'–3')	Application
SlMBP21-i	CGGGGTACCAAGCTTAATGGAGGACAAACAATGGAATAC<br>CCGCTCGAGTCTAGAGTCATAGTGCATAGTTGAGATGGG	To establish *SlMBP21* RNAi lines; add *Kpn*I+ *Hind* III and *Xho*I +*Xba*I site underlined, respectively
SlMBP21-full	AGAAAAAGTGTGTTAACCTAGAGTGG<br>ATGTCATAGTGCATAGTTGAGATGG	Full-length amplification of *SlMBP21*
SlMBP21 (Y2H)	CGCGGATCCTATGGGAAGAGGAAGAGTAGAACTA<br>AAAACTGCAGTTAGAGCATCCACCCTGGA	Construction of yeast two-hybrid vector
SFT (Y2H)	CCGGAATTCATGCCTAGAGAACGTGATCCTCTT<br>CGCGGATCCTCAATCAGCAGATCTTCTACGTCC	
MC (Y2H)	CCGGAATTCATGGGAAGAGGAAAAGTTGAATTA<br>CGCGGATCC TCATAGATGTTTATTCATGTTGTAAAGTG	
J (Y2H)	CCGGAATTCATGGCTAGAGAAAAAATTCAGATC<br>CGCGGATCCATCAGCCTGAGTAAGGTAGCCC	
SlCMB1 (Y2H)	CCGGAATTCATGGGAAGAGGTAAGGTAGAATTGA<br>CGCGGATCCTCAGATTCTGAATTCGCCCCT	
NPT II	CTCAGAAGAACTCGTCAAGAAGG<br>GACTGGGCACAACAGACAATC	Positive transgenic plants detection

Table S2. Specific primer sequences used for qRT-PCR analysis

Primer Name	Primer Sequence (5'–3')	Product (bp)
SlCAC	CCTCCGTTGTGATGTAACTGG<br>ATTGGTGGAAAGTAACATCATCG	173 bp
q-SlMBP21	GTTAGATCAAAAAAGACTCAATCTATGCT<br>TGTATTCCATTGTTTGTCCTCCAT	172 bp
q-SP	CAGACATTCCAGGCACTACAGAT<br>AACAGCAGCAACTGGTGAGC	212bp
q-STM3	TTGTTTATCGGACAACCTCATTAT<br>AACCCACTCATACATCTCCAAAA	207bp
q-JOINTLESS	AAGGCAACAGGTGATGGAGATA<br>GAGTAAGGTAGCCCCAATTTGAG	222bp
q-S	TCTTGCTACTACCACTATTCCCACC<br>CACCGAACACTTCCCTCACG	152bp
q-cycA2	TCGTCTCCAAAACCAGTCCAA<br>CGAAAGAGGGTTGAAAAGAATGT	174bp
q-cycD3	GTACCTGGTAGTCCAAGTGGTGTTA<br>GTAAACCATTGCCGAAATACTAGG	218bp
q-cdkB2	AGTGACAAACCAAGCCCTCTT<br>CATCAAGACCAGGGACAACG	179bp
q-E2FE	GCCTCCAGTTACCGTCCTCA<br>GGCAGTTGACTTCAATGTCCTG	169bp
q-cel2	ACACATTGCCAAACGTCAGGT<br>CCCCTATGGTGAATCCTTTGTG	108bp
q-XTH1	AGTATGAAAATGAGGCTTGTTGGT<br>AATGGTAGCCCTTGGTTGGA	217bp
q-EXPA18	CAGGAAGCAAGGTGGAATAAGG<br>CTAACCCTAAATGAAAGTGACTGACC	207bp
q-MAN1	CGATGATGCACAGTTGGTGTG<br>CCTGCCATTGTTCCACCTTC	195bp
q-LOG3	CGGTAGGATTGCTGAATGTGG<br>GCAGATACGATGATTTGACGAGC	112bp
q-LOG6	GCACGCCACATCATTGTATCA<br>TGTGCCTGAGGGTAACCAAGT	134bp
q-CKX2	GATACAGTAAATCAGGAAATTGAAGACT<br>TTTAGGGACTAGAAGATTGAGCCA	183bp
q-CKX7	CAAGAGAAGGGGTTATGGGATG<br>ATTTGTTGACAGGGTAGATGAGTAGAG	148bp
q-IAA2	GGTGCGGCTTATTTAAGGAAAA<br>GTCACCAGCAAGCATCCAATC	183bp
q-IAA5	GTGAATTTGAGTTCTCATATTTGCTA<br>CATCCAATCTCCATCTTTATCCT	180bp
q-ARF5	GGTGCATCATCAAGCAATGTAAA<br>CATTCTTTCAATTTCAGACCGC	183bp
q-ARF9	TGTTCCACCAGGTCTCGCAC<br>AATCACCCATAAGCATTGTATCCC	202bp
	AGAATAGTAATGAGTGGAGGAGCAA	

q-ACS1A	ATTACAACTTTCACAAACAACTGGA	171bp
q-ACS6	TGATCCTGGTGATGCATTTCTAGTTC<br>CTTCTTCTAAGGCTTCTTTTGTTACC	146bp
q-ERF-H1	AGTAGTCAACTTGGGAGTGGGA<br>CACTGGATTTGGTGAGAAGGA	177bp
q-ERF5	CGAAGTCGTCAGGCAGTAAAAG<br>TGAGATAATGGTGACAATGGTGG	182bp

Supplementary Figures

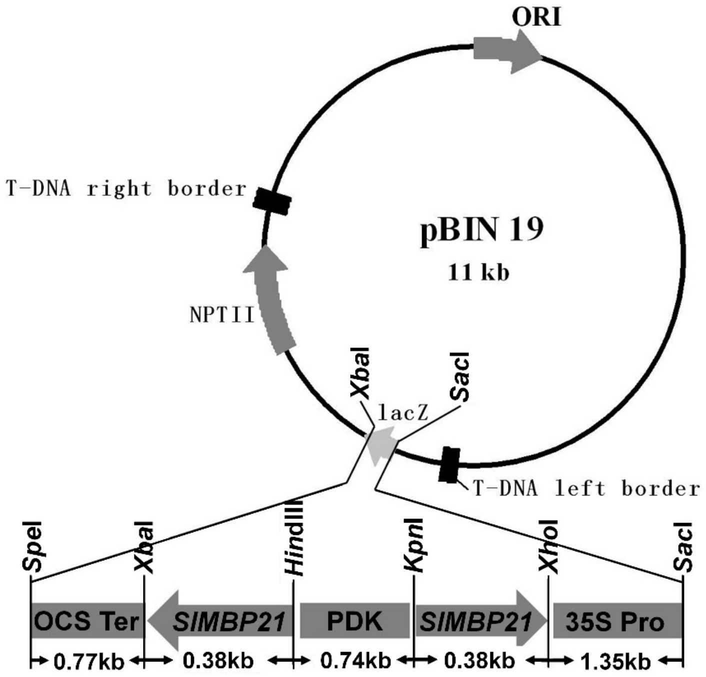

Figure S1 The construction of *SlMBP21*-RNAi vectors

Hairpin construct of the *SlMBP21* gene for double-stranded RNAi vector. The *SlMBP21* gene-specific sequence in the antisense and sense orientations were linked with a PDK gene fragment and as a transcriptional unit for hairpin RNA expression which premoted by the CaMV 35S promoter and terminated by the OCS terminator. Among which, *Spe*I and *Xba*I are isocaudamers.

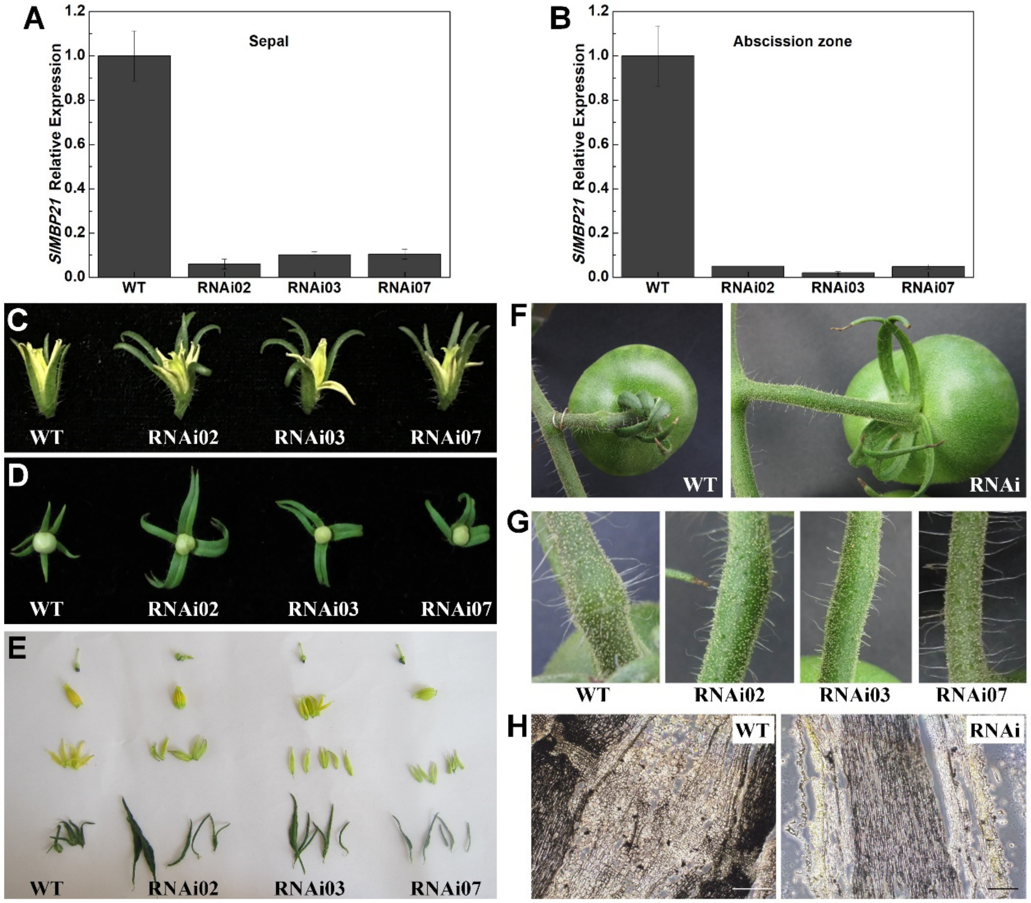

Figure S2 The Phenotype of sepals and abscission zone in *SlMBP21* Repressed Lines.

(A). The relative expression of *SlMBP21* in WT and *SlMBP21*-RNAi lines. The tissue examined was sepals. Expression values are relative to the *SlCAC* gene. (B). The relative expression of *SlMBP21* in WT and *SlMBP21*-RNAi lines. The tissue examined was abscission zone. Expression values are relative to the *SlCAC* gene. (C-E). Phenotypes of sepals in WT and *SlMBP21*-RNAi lines. (F-G). Phenotypes of abscission zone in WT and *SlMBP21*-RNAi lines. (H). Light micrographs showing the fruit pedicel abscission zone in WT and *SlMBP21*-RNAi lines. Bars = 100 μm.

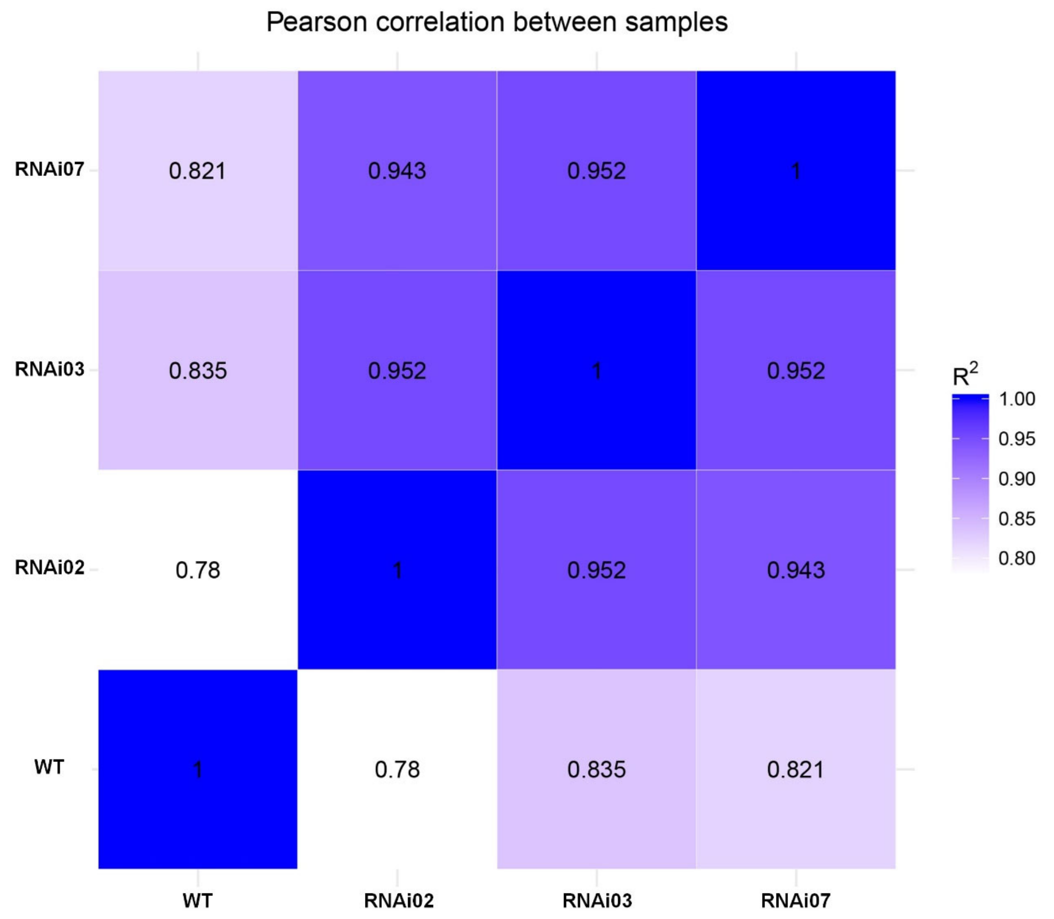

Figure S3 Correlation analysis (through Pearson's correlation coefficient) of RNA-seq data among WT, RNAi02, RNAi03, RNAi07.

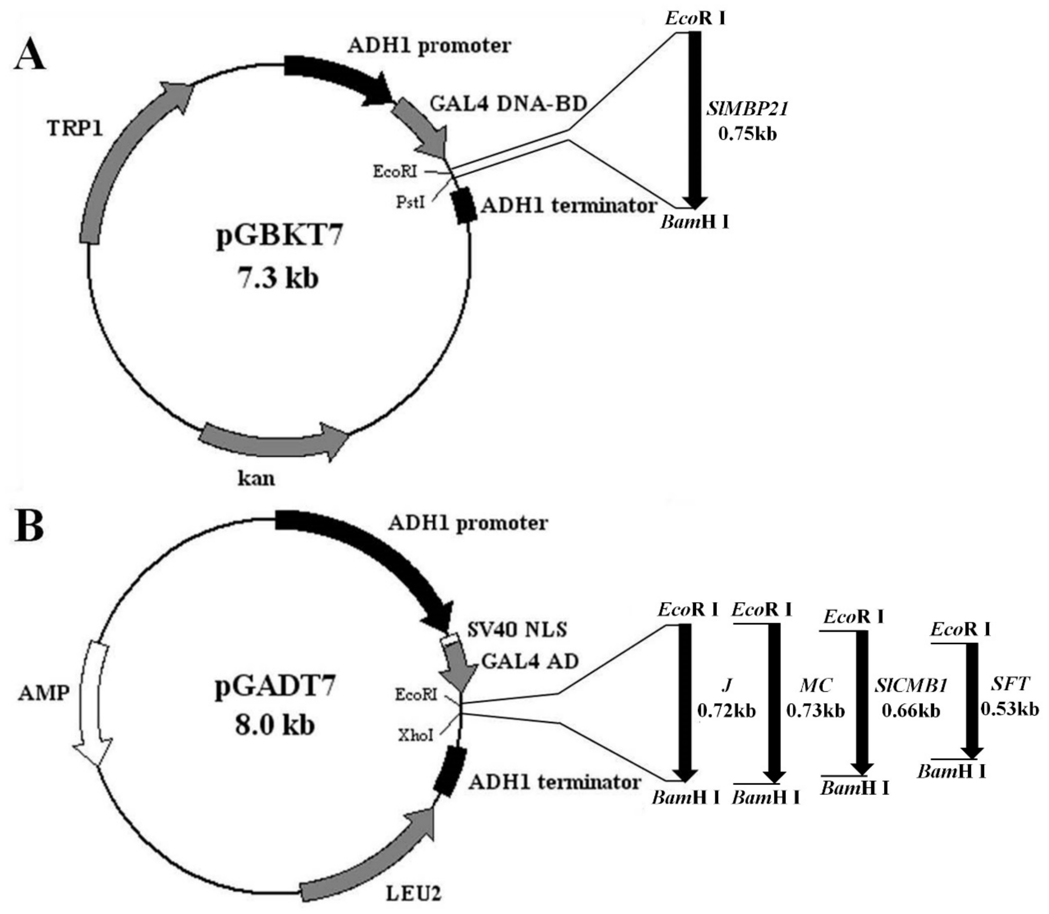

Figure S4 Construct of *SlMBP21*, *JOINTLESS*, *MC*, *SlCMB1* and *SFT* gene for the yeast two-hybrid vector.

(A). The ORFs of *SlMBP21* was cloned into pGBKT7 bait vector to obtain the vector pGBKT7-*SlMBP21*, respectively. (B). The ORFs of *JOINTLESS*, *MC*, *SlCMB1* and *SFT* were cloned into pGADT7 prey vector to obtain the vector pGADT7-*JOINTLESS*, pGADT7-*MC*, pGADT7-*SlCMB1* and pGADT7-*SFT*.

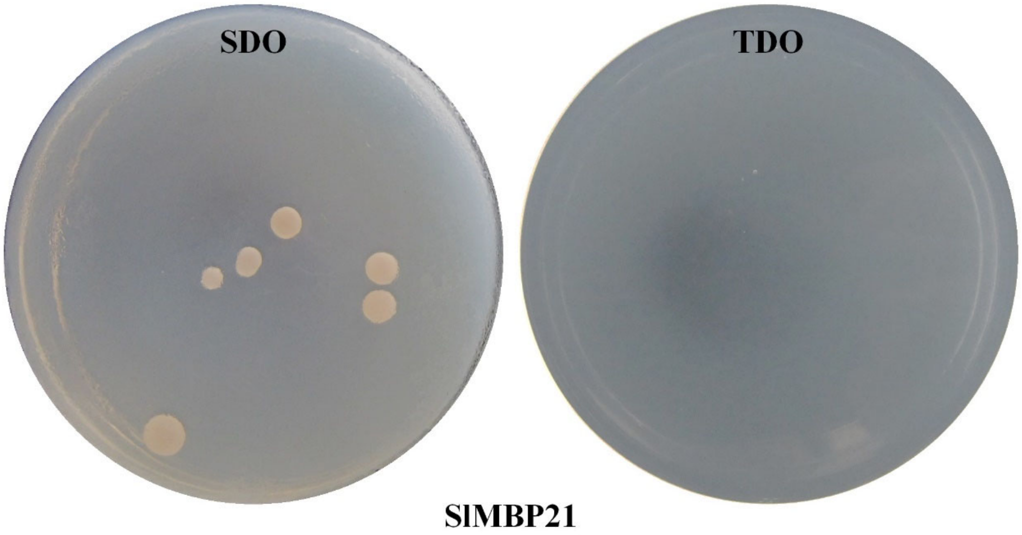

Figure S5 Self-activation assay of pGBKT7-*SlMBP21* in the yeast two-hybrid assay.

Yeasts with pGBKT7-*SlMBP21* were plate on SDO and TDO medium. All these two yeasts had no self activation, they can grow on the SDO medium but can not grow on the TDO medium, respectively. TDO, SD medium without Trp, His, Ade; SDO, SD medium without Trp.
