# Supplementary figures and images for: A SEPALLATA MADS-Box Transcription Factor, SlMBP21, Functions as a Negative Regulator of Flower Number and Fruit Yields in Tomato
Source: Plants (Basel). 2024 May 20;13(10):1421. doi: 10.3390/plants13101421 (PMC11125064; doi:10.3390/plants13101421)

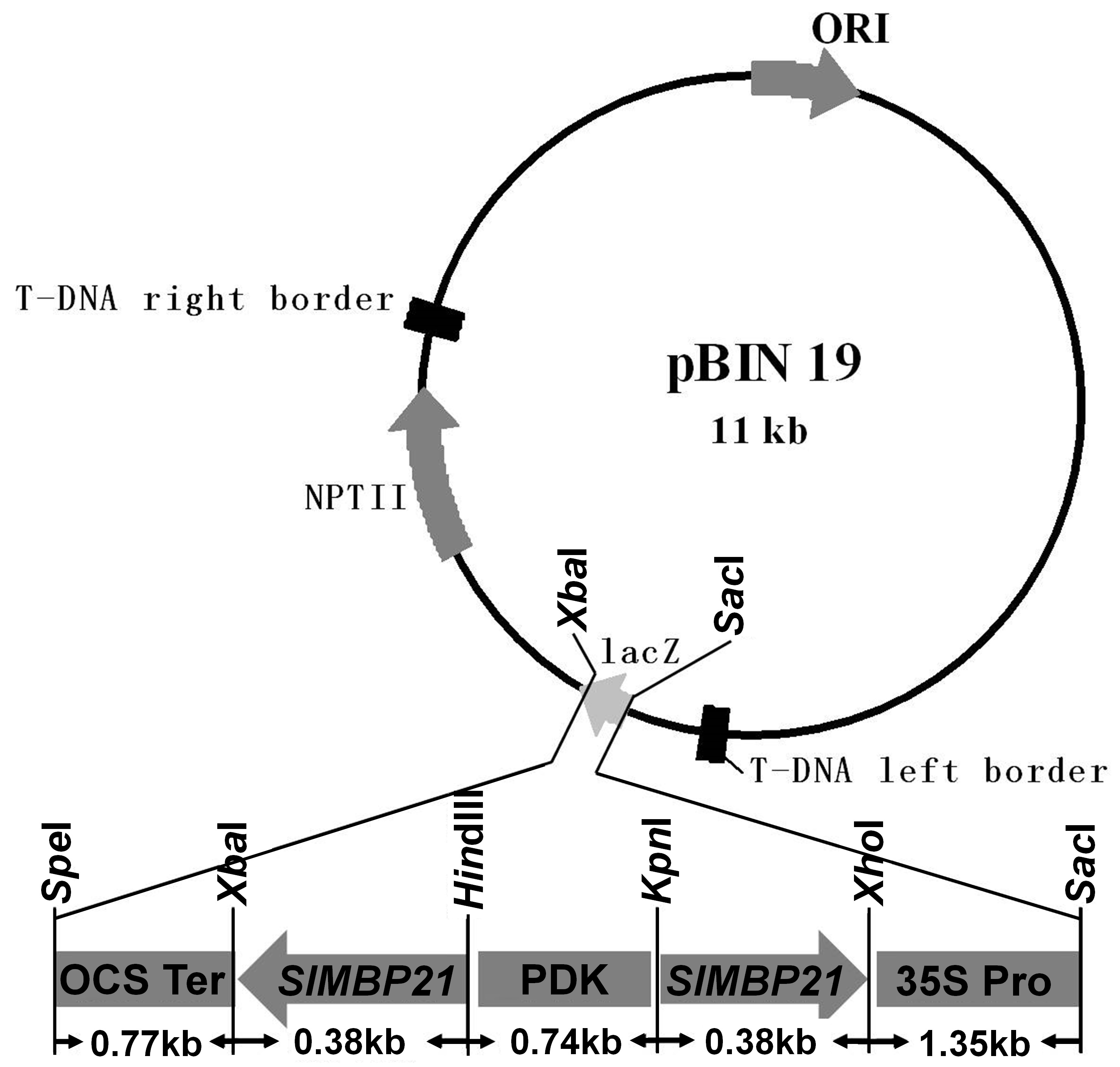

Supplement: Supplementary file 1 [file plants-13-01421-s001.zip › Figure S1.jpg]

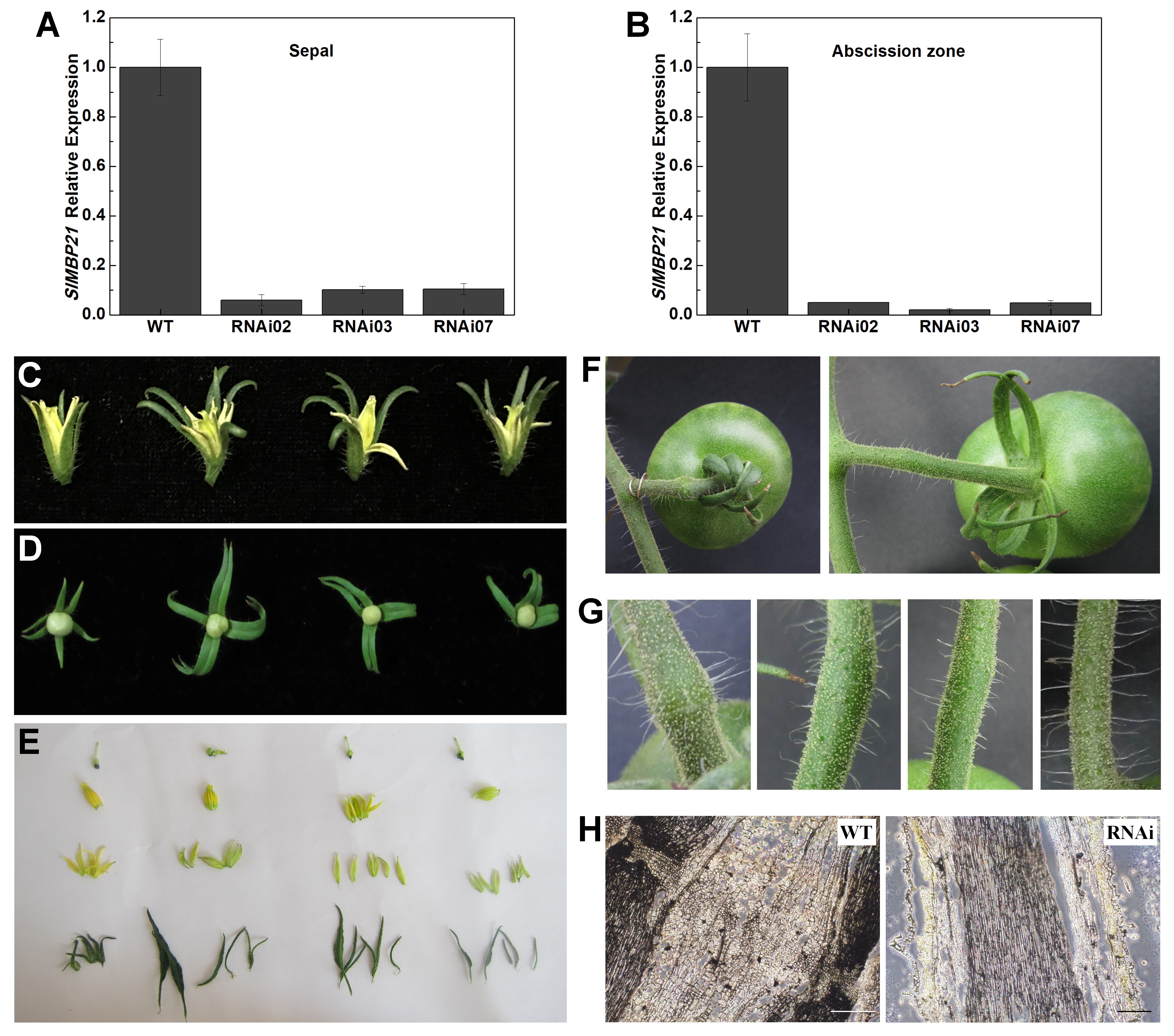

Supplement: Supplementary file 1 [file plants-13-01421-s001.zip › Figure S2.jpg]

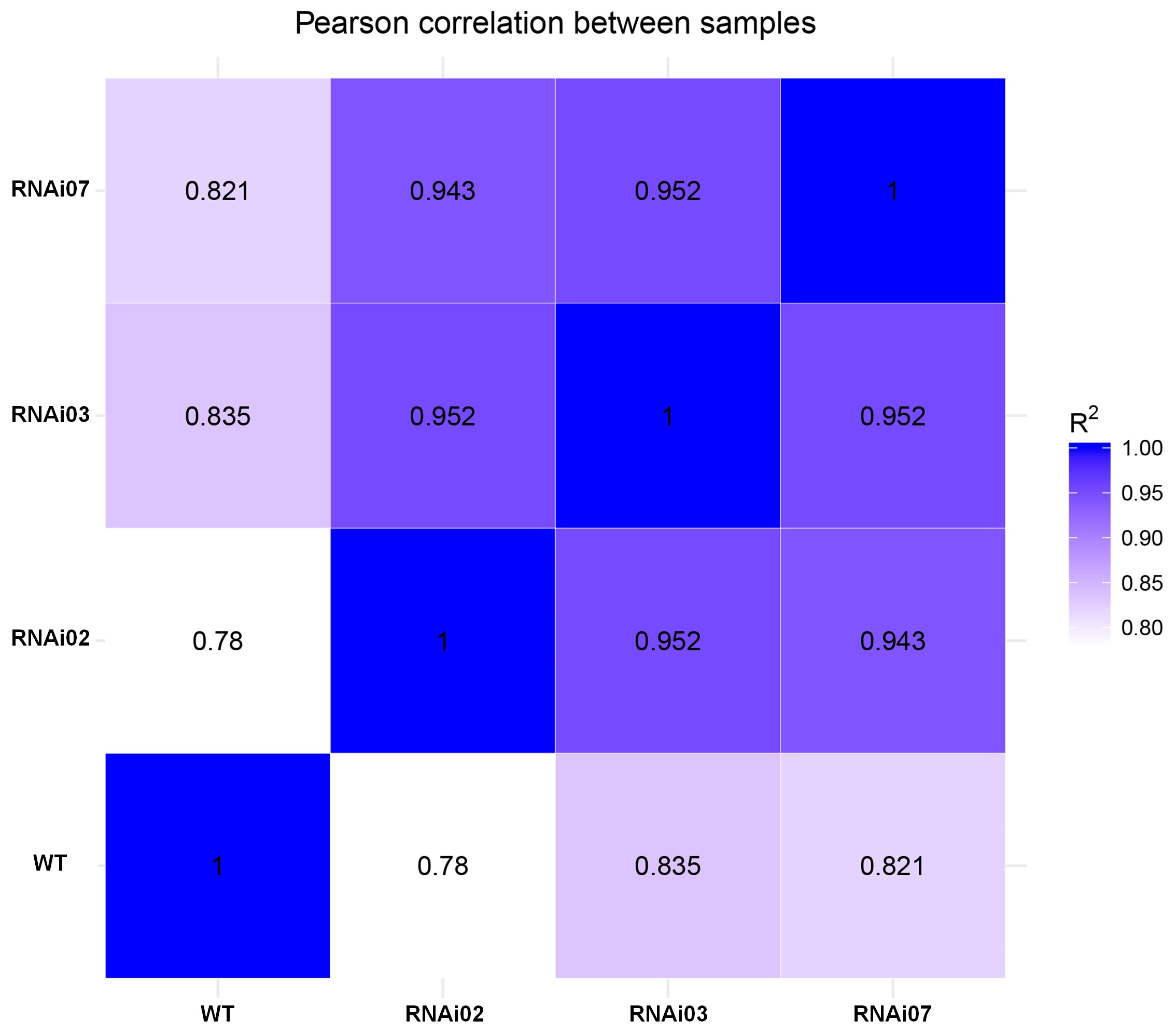

Supplement: Supplementary file 1 [file plants-13-01421-s001.zip › Figure S3.jpg]

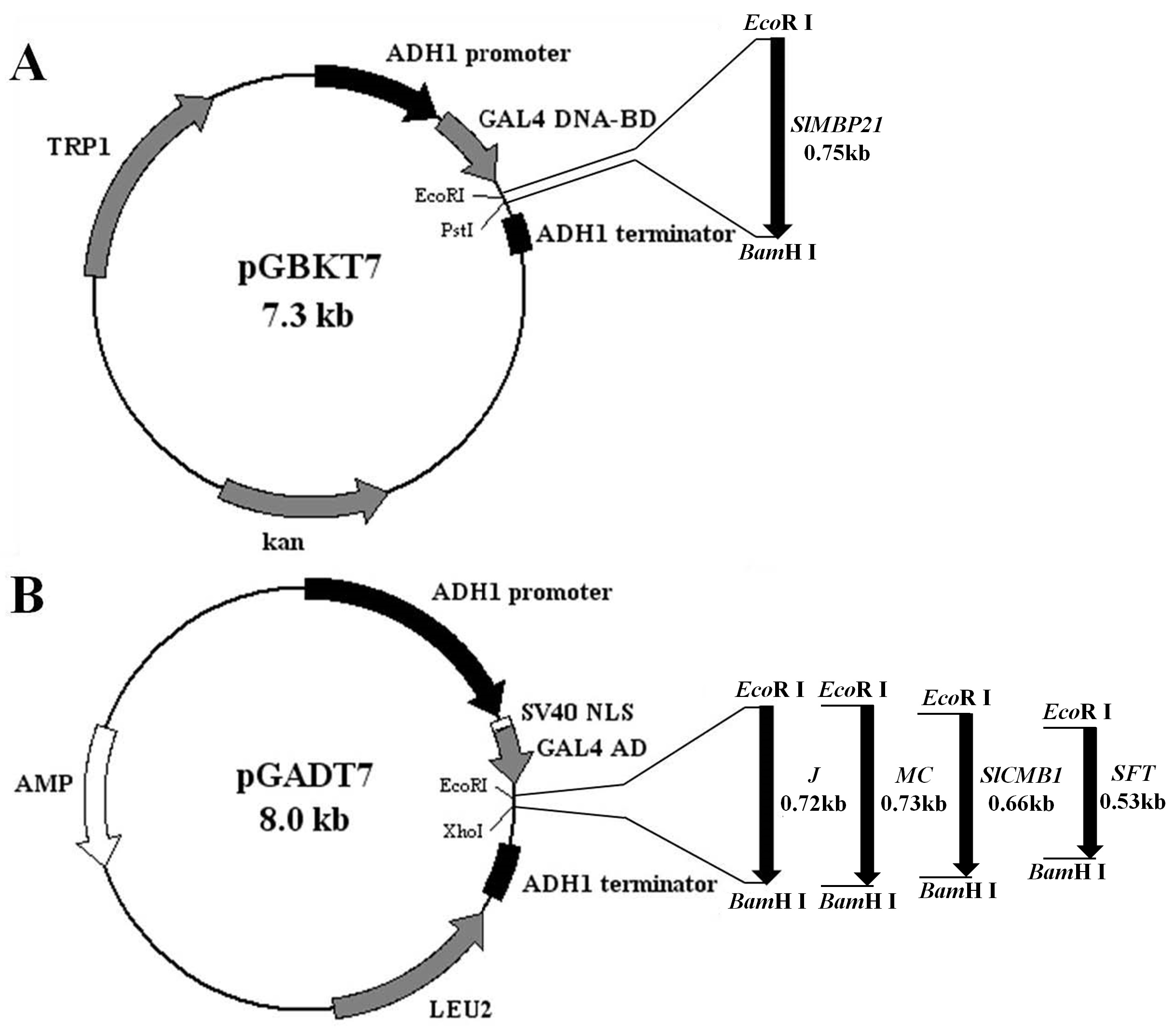

Supplement: Supplementary file 1 [file plants-13-01421-s001.zip › Figure S4.jpg]

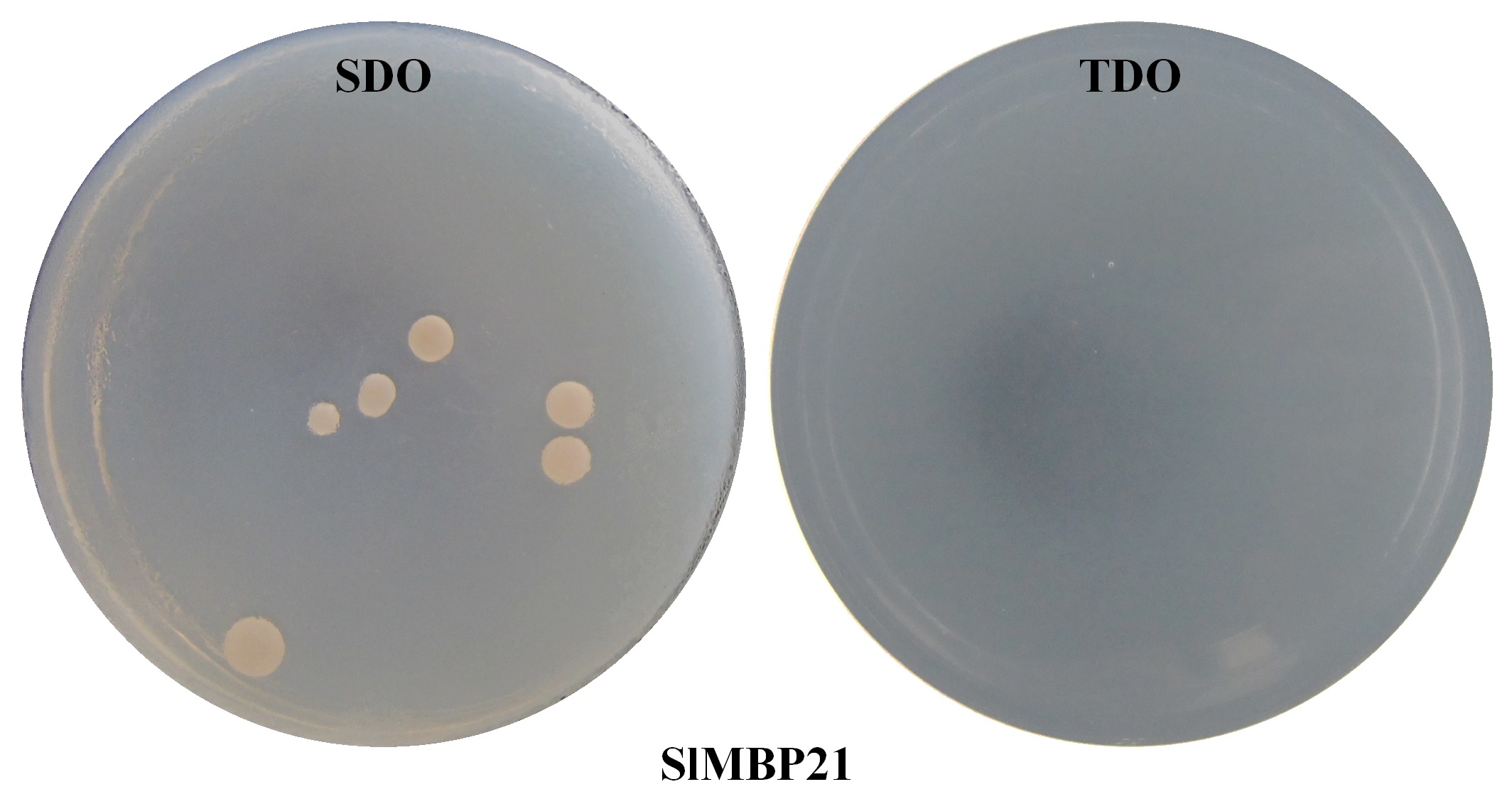

Supplement: Supplementary file 1 [file plants-13-01421-s001.zip › Figure S5.jpg]
